# Clinical Behaviour and Outcome of Pelvic Osteosarcoma in Dogs: A Retrospective Multicentre Analysis of 74 Cases (2007–2025)

**DOI:** 10.1111/vco.70106

**Published:** 2026-08-22

**Authors:** Csilla Fejös, Felicitas Czichon, Maureen Griffin, Natalie Grillo, Quentin Fournier, Michelle L. Oblak, Mikaela Eng‐Toogood, Michael Kent, Isabelle Desmas‐Bazelle, Miriam Kleiter, Laura Marconato, Katie McNaught, Simona Vincenti, Carmit Chalfon, Petros Odatzoglou, Carla Rohrer Bley

**Affiliations:** ^1^ Clinic for Radiation Oncology & Medical Oncology, Small Animal Department Vetsuisse Faculty, University of Zurich Zurich Switzerland; ^2^ Department of Clinical Sciences Colorado State University College of Veterinary Medicine and Biomedical Sciences Fort Collins Colorado USA; ^3^ Aura Veterinary Guildford UK; ^4^ Lumbry Park Veterinary Specialists Alton UK; ^5^ Ontario Veterinary College, University of Guelph Guelph Canada; ^6^ Department of Surgical and Radiological Sciences School of Veterinary Medicine, University of California‐Davis Davis USA; ^7^ Department of Clinical Science and Services Royal Veterinary College, University of London London UK; ^8^ Clinical Department of Small Animals and Horses University of Veterinary Medicine Vienna Vienna Austria; ^9^ Department of Veterinary Medical Sciences University of Bologna Bologna Italy; ^10^ Small Animal Clinical Sciences, Small Animal Hospital, University of Glasgow Glasgow UK; ^11^ Department for Clinical Veterinary Science Vetsuisse Faculty, University of Bern Bern Switzerland; ^12^ Centro Veterinario Torinese Torino Italy; ^13^ Queen's Veterinary School Hospital, Department of Veterinary Medicine University of Cambridge Cambridge UK

**Keywords:** canine, chemotherapy, pelvis, radiation therapy, surgery

## Abstract

Although osteosarcoma is the most common primary bone tumour in dogs, involvement of the pelvic bones is rare. The aim of the present study was to describe clinical presentation, identify possible prognostic factors, and evaluate outcomes in dogs diagnosed with pelvic osteosarcoma. Seventy‐four dogs with cytologically or histologically diagnosed pelvic osteosarcoma were included in this multicentre retrospective study (2007–2025). The median progression‐free survival (PFS) and overall survival (OS) were estimated using the Kaplan–Meier method, with comparisons performed using log‐rank tests. Prognostic factors for PFS and OS were assessed using multivariable Cox proportional hazards regression analysis. Lameness (84%) was the most common presenting clinical sign, and the ilium (57%) was the most frequent intrapelvic tumour location. Thirty‐one percent of dogs had metastases at diagnosis, primarily affecting the lungs. The median PFS was 75 days, and the median OS was 79 days. Dogs receiving any treatment had significantly longer PFS (116 vs. 7 days, *p* < 0.001) and OS (145 vs. 8 days, *p* < 0.001) than untreated dogs. No factors were significantly associated with PFS. In contrast, dogs with higher body weight and shorter time to diagnosis tended to have more favourable survival, whereas chemotherapy and local progression were associated with poorer outcomes. Despite the overall poor prognosis associated with pelvic osteosarcoma, treatment may extend survival. An individualized, often multimodal approach may provide clinical benefit in selected dogs.

**Abbreviations**
ALP: alkaline phosphatase
CI: confidence interval
CT: computed tomography
HPF: high‐power field
HR: hazard ratio
IQR: interquartile range
LN: lymph node
MC: mitotic count
MRI: magnetic resonance imaging
NK: natural killer
OS: overall survival
PFS: progression‐free survival
RT: radiation therapy
SBRT: stereotactic body radiation therapy
SD: standard deviation
TTDP: time to distant progression
TTLP: time to local progression

## 1 Introduction

Osteosarcoma accounts for approximately 85% of all primary bone tumours in dogs and is characterized by aggressive biological behaviour and a high metastatic potential, particularly to the lungs [1, 2]. While appendicular locations predominate, tumours originating from axial, flat, and irregular bones are less frequent. Osteosarcoma arising from the pelvic bones is particularly rare, representing only 1%–2% of all cases [3].

Although anatomically part of the appendicular skeleton, the pelvis is frequently classified as an axial location, a distinction likely linked to its structural and functional characteristics [3, 4, 5, 6, 7]. Pelvic bones lack a traditional medullary cavity and consist mainly of cortical plates surrounding the inner trabecular tissue, serving primarily as support structures rather than levers for locomotion [6, 7]. These anatomic differences suggest that pelvic osteosarcoma may behave differently from those in more typical appendicular sites and share features with axial bone tumours.

In humans, osteosarcoma with a pelvic location is considered particularly aggressive, with high rates of metastases at diagnosis (26%–34%) and poorer outcomes than those of long bone tumours, attributed in part to the patient's older age, large tumour size, delayed diagnosis, difficulty of achieving wide surgical margins, and overall lower surgical resection rate [8, 9, 10]. In dogs, the metastatic prevalence at diagnosis appears to be lower (5%–15% for appendicular and 11%–18% for axial tumours) than in people; however, prognosis remains poor, as most dogs with pelvic or axial tumours, except for the mandibular cases, are euthanized due to complications related to the primary tumour [3, 4, 11, 12, 13, 14, 15, 16, 17, 18]. As in humans, the extent of the disease and complex pelvic anatomy, including proximity to major neurovascular and visceral structures, make hemipelvectomy a demanding procedure [19].

Due to these challenges, radiation therapy (RT) – more often hypofractionated– has alternatively been recommended for axial, flat, and irregular bones, despite the known relative radioresistance of osteosarcoma [3, 4, 18].

Several prognostic factors have been investigated in canine appendicular and axial osteosarcoma, with elevated serum alkaline phosphatase (ALP) and the humeral location being consistently associated with poorer outcomes [20, 21, 22, 23, 24]. However, there is currently no robust evidence to suggest that pelvic bone involvement independently confers a distinct prognostic significance—favourable or unfavourable—in affected dogs [16, 20, 21, 22, 23, 24].

Given the limited information available regarding pelvic osteosarcoma in dogs, this study aimed to retrospectively evaluate a larger, anatomic site‐specific cohort by describing dog characteristics, clinical presentation, treatment modalities, and outcomes.

## 2 Materials and Methods

### 2.1 Case Data

Medical records between 2007 and 2025 from 12 veterinary referral institutions were reviewed. Dogs were eligible for inclusion if they had cytologically suspected and/or histologically confirmed osteosarcoma involving any component of the pelvis.

Dogs with additional bony lesions were included when primary clinical signs and treatment were directed toward the pelvic lesion. Dogs were excluded if osteosarcoma was suspected based solely on diagnostic imaging.

### 2.2 Data Collection

Data obtained from the medical records included signalment (age, breed, sex, body weight), presenting clinical signs and their duration, pelvic tumour location, the diagnostic modalities used for diagnosis and staging (radiography, ultrasound, computed tomography (CT), magnetic resonance imaging (MRI), cytology with or without ALP staining, and/or histology) and haematologic parameters and serum ALP when available. Information on local (surgery, RT), systemic (chemotherapy), and palliative (bisphosphonates, analgesics) treatments when performed, disease progression (local and/or distant), cause of death (tumour‐related, unrelated, or unknown), necropsy results when performed, and follow‐up were collected. Referring veterinarians were contacted when necessary to complete follow‐up.

When available, histology results included osteosarcoma subtype, mitotic count, histological margin status (complete or incomplete), and tumour grade [25].

Local progression was defined as worsening of initial clinical signs attributable to the primary tumour or radiologic evidence of tumour growth in dogs without treatment, systemic treatment, and RT, or as recurrence at the surgical site for surgically treated dogs. Distant progression was defined on imaging as newly detected distant lesions consistent with osteosarcoma metastasis, with or without cytologic/histologic confirmation.

Surgery‐based treatment was defined as surgical excision of the primary tumour, regardless of adjunctive therapy. RT‐based treatment included definitive‐ or palliative‐intent RT, as a local treatment, with or without additional therapy.

### 2.3 Statistical Analysis

Descriptive statistics were used to characterize all variables. Categorical data were reported as numbers and percentages, and continuous variables were assessed for distribution using histograms and measures of skewness and kurtosis, then presented as means ± standard deviation (SD) or medians with ranges or interquartile ranges (IQR), as appropriate.

Progression‐free survival (PFS) was defined as the interval from diagnosis to the first documented disease progression or death from any cause, and overall survival (OS) was defined as the interval from diagnosis to death from any cause. Time to local progression (TTLP) and time to distant progression (TTDP) were defined as intervals from diagnosis to local tumour growth/recurrence and development of the first metastatic lesion, respectively. Kaplan–Meier methods were used to estimate all time‐to‐event endpoints. The log‐rank test was used to assess differences in PFS and OS between treated and untreated dogs. Dogs that died without documented progression were considered to have experienced a PFS event. Dogs still alive or lost to follow‐up were censored at last follow‐up.

PFS and OS were also evaluated within predefined treatment subgroups (surgery‐based treatment, RT‐based treatment, palliative RT, and SBRT). Furthermore, TTLP and TTDP were assessed for all dogs, all treated dogs, and the surgery‐based and RT‐based cohorts. These analyses were performed descriptively to provide clinical context; no statistical comparisons were performed due to small sample sizes and overlapping treatment categories.

Cox proportional hazards regression was performed for treated dogs to identify factors associated with PFS and OS.

Variables assessed included body weight, time to diagnosis, ALP activity, iliac tumour location, metastasis at diagnosis, and the use of RT, surgery, and chemotherapy. Local and distant progression were additionally included in the survival model. Variables were tested for multicollinearity, and manual backward selection was applied to the multivariable models by sequentially removing the variable with the highest *p*‐value to maintain approximately 10 events per variable and avoid overfitting; significance was set at *p* < 0.05. Analyses were performed using R Core Team and IBM SPSS Statistics (Version 30).

## 3 Cell Line Validation Statement

No cell lines were used in the current study.

## 4 Results

### 4.1 Signalment, Clinical Signs, and Tumour Locations

A total of 74 dogs with pelvic osteosarcoma met the inclusion criteria (Table 1). The cohort consisted predominantly of older dogs, with a median age of 10 years (IQR, 8–11), and had a mean body weight of 32 ± 14 kg. Clinical signs had a median duration of 19 days (IQR, 14–57). Among the 11 dogs (15%) showing signs of pelvic compression, four presented with no other clinical signs, whereas the remaining dogs (*n* = 7) had additional findings.

**TABLE 1:** Baseline characteristics of 74 dogs with pelvic osteosarcoma.

Case demographics	*n*/*N* (%)
*Sex and neuter status*	
Male intact	11/74 (15)
Male neutered	35/74 (47)
Female intact	4/74 (5)
Female spayed	24/74 (32)
*Body weight categories*	
< 15 kg	7/74 (9)
15–39 kg	46/74 (62)
> 40 kg	21/74 (28)
*Breed*	
Mixed breed	21/74 (28)
Labrador Retriever	9/74 (12)
Golden Retriever	5/74 (7)
Rottweiler	5/74 (7)
Bernese Mountain Dog	2/74 (3)
Rhodesian Ridgeback Chesapeake Bay	2/74 (3)
Retriever	2/74 (3)
Greyhound	2/74 (3)
Cocker Spaniel	2/74 (3)
24 other breeds	24/74 (32)
*Clinical signs at presentation*	
Lameness	62/74 (84)
Dyschezia	11/74 (15)
Pain	7/74 (9)
Palpable mass	4/74 (5)
Lethargy	3/74 (4)
Rectal mass effect	3/74 (4)
Dysuria	3/74 (4)
Uncommon signs^a^	≤ 2/74 (< 3)
*Pelvic location*	
Single sites	
Ilium	42/74 (57)
Ischium	11/74 (15)
Sacrum	5/74 (7)
Pubis	3/74 (4)
Acetabulum	2/74 (3)
Symphysis	1/74 (1)
Multifocal/combined sites	
Ilium and sacrum	5/74 (7)
Ilium and soft tissue	2/74 (3)
Ilium and acetabulum	1/74 (1)
Acetabulum and pubis	1/74 (1)
Unspecified	1/74 (1)

Abbreviations: *n*, number; *N*, total number.

^a^ Uncommon clinical signs included polyuria and polydipsia (*n* = 2), urinary obstruction (*n* = 2), tremor (*n* = 1), muscle atrophy (*n* = 1), inappetence (*n* = 1), and vomiting (*n* = 1).

### 4.2 Tumour Diagnosis and Staging

Pelvic imaging was available for 73/74 (99%) dogs. In 1/74 (1%) dog, imaging was not performed due to suspected lung metastasis; however, osteosarcoma was confirmed histologically at necropsy. CT was performed in 34/73 (47%), radiography in 16/73 (22%), both modalities in 17/73 (23%), and MRI in 5/73 (7%); two dogs (3%) had pelvic ultrasound in addition to other imaging. The diagnosis was suspected cytologically in 33/74 (45%), confirmed histologically in 28/74 (39%), and established by both methods in 13/74 (18%) cases. ALP staining was performed on 14/46 (30%) cytologic samples. Histological findings are summarized in Table 2. The median mitotic count was 14 mitoses per 10 high‐power fields (equivalent to 2.37 mm^2^).

**TABLE 2:** Histological results of dogs with pelvic osteosarcoma.

	*n*/*N* (%)
Histological subtype	29/41 (71)
Osteoblastic	16/29 (55)
Chondroblastic	6/29 (21)
Fibroblastic	2/29 (7)
Giant cell	2/29 (7)
Mixed^a^	3/29 (10)
Histologic grade	3/41 (7)
Grade II	1/3 (33)
Grade III	2/3 (67)
Mitotic count	23/41 (56)
Surgical margins	15/17 (88)
Complete	5/15 (33)
Incomplete	10/15 (67)

Abbreviations: *n*, number affected or assessed; *N*, total number.

^a^ Mixed subtypes included fibroblastic and giant cell (*n* = 1 each), osteoblastic and chondroblastic (*n* = 1), and osteoblastic and telangiectatic (*n* = 1).

Hematologic data were available for 63/74 (85%) dogs. Anaemia was present in 13/63 (21%; haematocrit range 29.7%–39%; reference interval (RI), 42%–55%), neutrophilia in 30/63 (48%; range, 7.7–22.4 × 10^9^/L; RI, 2.5–7.4 × 10^9^/L), monocytosis in 14/63 (22%; range, 1.1–2.3 × 10^9^/L, RI, 0.2–0.9 × 10^9^/L), and lymphopenia in 15/63 (24%; range 0.4–1.0 × 10^9^/L; RI, 1.2–3.4 × 10^9^/L). Serum ALP was available in 64/74 (86%) dogs and was increased in 26/64 (41%), with values ranging from 102 to 5594 U/L (RI, 20–98 U/L). Thoracic imaging was performed in 71/74 (96%) dogs, using radiography in 35 (49%), CT in 35 (49%), and both in 1 (1%). Abdominal imaging was available in 49/74 (66%) dogs, consisting of CT in 31 (63%), ultrasound in 6 (12%), and both in 2 (4%). Metastases were identified on initial presentation or staging in 23/74 (31%) dogs, most commonly in the lungs (15/23, 65%). Other sites included lymph nodes (LN; *n* = 8; medial iliac (*n* = 5), medial iliac and hypogastric (*n* = 2), and inguinal (*n* = 1)), vertebral body (*n* = 2), femoral diaphysis (*n* = 1), ilium (*n* = 1), radius (*n* = 1), rib (*n* = 1), intercostal space (*n* = 1), and kidney (*n* = 1). Multiple metastatic sites were present in 7 (30%) dogs. Metastases were cytologically confirmed in two dogs (9%; LNs, lungs, and kidney) and histologically confirmed in the lungs of an additional two dogs (9%).

### 4.3 Treatment

Forty‐five of 74 (61%) dogs received definitive treatment, consisting of either single‐modality (28/45; 62%) or a multimodal approach (17/45; 38%) that included surgery, RT, and/or chemotherapy (Table 3). Twenty‐nine dogs (39%) received palliative care using bisphosphonates and/or analgesics. Fifteen of the 45 (33%) treated dogs had presumptive or confirmed metastases at diagnosis, compared with 8/29 (28%) untreated dogs.

**TABLE 3:** Overview of treatment modalities in dogs with pelvic osteosarcoma.

	*n*/*N* (%)	Local progression *n*/*N* (%)	Distant progression *n*/*N* (%)	No known progression *n*/*N* (%)	Lost to follow‐up *n*/*N* (%)
Treatment	45/74 (61)	30/45 (65)	13/45 (28)	6/45 (13)	2/45 (4)
Single‐modality treatment					
RT	21/45 (46)	15/21 (71)	5/21 (24)	1/21 (5)	2/21 (10)
Surgery	5/45 (11)	1/5 (20)	1/5 (20)	3/5 (60)^a^	—
Chemotherapy	2/45 (4)	2/2 (100)	2/2 (100)		
Multimodal treatment					
Surgery and chemotherapy	9/45 (22)	7/9 (70)	1/9 (20)	1/9 (10)	—
RT and chemotherapy	5/45 (11)	3/5 (60)	3/5 (60)	1/5 (20)	—
Surgery, RT, and chemotherapy	2/45 (4)	2/2 (100)	—	—	—
Surgery and RT	1/45 (2)	—	—	–^a^	—
No treatment	29/74 (39)	15/29 (50)	1/29 (3)	6/29 (23)^b^	8/29 (27)

Abbreviations: *n*, number affected or assessed; *N*, total number; RT, radiotherapy.

^a^ Alive and/or without known progression.

^b^ Six dogs euthanized at diagnosis.

#### 4.3.1 Surgery

Seventeen out of 45 (38%) dogs underwent surgery: 12/17 (71%) external (removal of a pelvic segment along with the ipsilateral limb) and 2/17 (12%) internal (limb‐sparing, consisting of one cranial (iliac) and one caudal (ischial)) hemipelvectomies, and three (18%) cytoreductive surgeries. Three of 17 dogs (18%) were treated despite presumptive metastatic disease, including one that received postoperative dasatinib.

Of the dogs with metastases, two were euthanized due to local progression, and one due to widespread metastatic disease and local recurrence. Additionally, four dogs were euthanized shortly after surgery (range, 10–39 days) due to poor recovery or difficulty coping with amputation.

#### 4.3.2 Radiation Therapy

Twenty‐nine of 45 (64%) dogs received RT. Most (28/29, 97%) were treated with hypofractionated protocols: 20/29 (69%) palliative‐intent and 8/29 (28%) SBRT; RT details were unavailable for one dog.

For palliative RT, the median total dose was 20 Gy (range, 16–36 Gy) delivered in a median of 4 fractions (range, 2–8) with a median fraction size of 8 Gy (range, 3.5–10 Gy). One dog (5%) received 30 Gy (5 × 6 Gy) with a central boost of 36 Gy (5 × 7.2 Gy), and another (5%) underwent a quad‐shot protocol (4 fractions in 48 h); details were missing for two dogs (10%). For SBRT, the median total dose was 33 Gy (range, 30–36 Gy), delivered in a median of 3 fractions (range, 3–5), with a median fraction size of 11 Gy (range, 6–12 Gy).

Among the dogs with metastases at diagnosis (9/29, 31%), six received palliative RT and three SBRT, with one receiving additional chemotherapy (carboplatin). Four of these dogs were euthanized due to their local progression, and three to distant progression, while two dogs were lost to follow‐up.

#### 4.3.3 Systemic Treatment

Adjuvant maximal tolerated dose chemotherapy was administered to 14/18 (78%) dogs, consisting of carboplatin (*n* = 13) and cyclophosphamide (*n* = 1).

Seven received chemotherapy post‐operatively, four following SBRT, one following palliative RT, and two in conjunction with both surgery and palliative RT. Only 4/14 (29%) dogs completed the intended protocol (three with carboplatin and one with cyclophosphamide); 7/14 (50%) were euthanized after a median of one cycle (range, 1–4; seven receiving carboplatin) due to disease progression (6 with local progression, 1 with distant progression). Two dogs discontinued treatment after the first and third cycles of carboplatin, respectively, due to adverse effects. For one dog receiving carboplatin, records were unavailable. Additional systemic therapies included adjuvant dasatinib (1/18; 6%) and intratumoral autologous NK cell/IL‐15 immunotherapy (1/18, 6%), as well as metronomic chemotherapy (2/18, 11%; cyclophosphamide, thalidomide, and piroxicam) for lung metastases.

#### 4.3.4 Bisphosphonates

Sixteen of 74 (16/74; 22%) dogs received palliative treatment with bisphosphonates, specifically pamidronate (*n* = 8), zoledronate (*n* = 6), and alendronate (*n* = 2). Bisphosphonates were administered in combination with palliative RT in nine dogs (56%), used as monotherapy in six dogs (38%), and given to one dog (6%) using alendronate after surgery and chemotherapy, once carboplatin was discontinued due to adverse effects.

### 4.4 Disease Progression and Clinical Outcome

Progressive disease was documented in 51/74 (69%) dogs, including 38 (51%) with local progression, 6 (8%) with distant progression, and 7 (9%) with both (Table 3). Thirteen dogs developed new metastatic lesions; five of these already had metastases at diagnosis, with eight dogs (12%) developing *de novo* metastases. Combined with the 23 dogs that had metastases at diagnosis, this resulted in an overall metastatic rate of 42% (31/74). Newly affected sites included the lungs (*n* = 3), ribs (*n* = 2), sacrum (*n* = 1), subcutaneous tissue (*n* = 1), lungs and skin (*n* = 1), spleen and lung (*n* = 1), and abdominal LNs (*n* = 1). Pathologic fractures occurred in one dog with systemic treatment and three with palliative RT. Metastases were confirmed cytologically (*n* = 1, cutaneous lesions) or via necropsy (*n* = 2, lungs, abdominal LNs).

Among the 45 treated dogs, 36 (80%) developed progressive disease, including 25 (56%) with local, 6 (13%) with distant, and 5 (11%) with both (Table 3). When the five dogs with pre‐existing metastases were excluded, 7/40 (18%) developed *de novo* metastases post‐treatment.

Median PFS for all dogs was 75 days (95% CI, 50–100), with a longer median PFS observed in treated than untreated dogs (*p* < 0.001) (Figure 1, Table 4).

**FIGURE 1:**
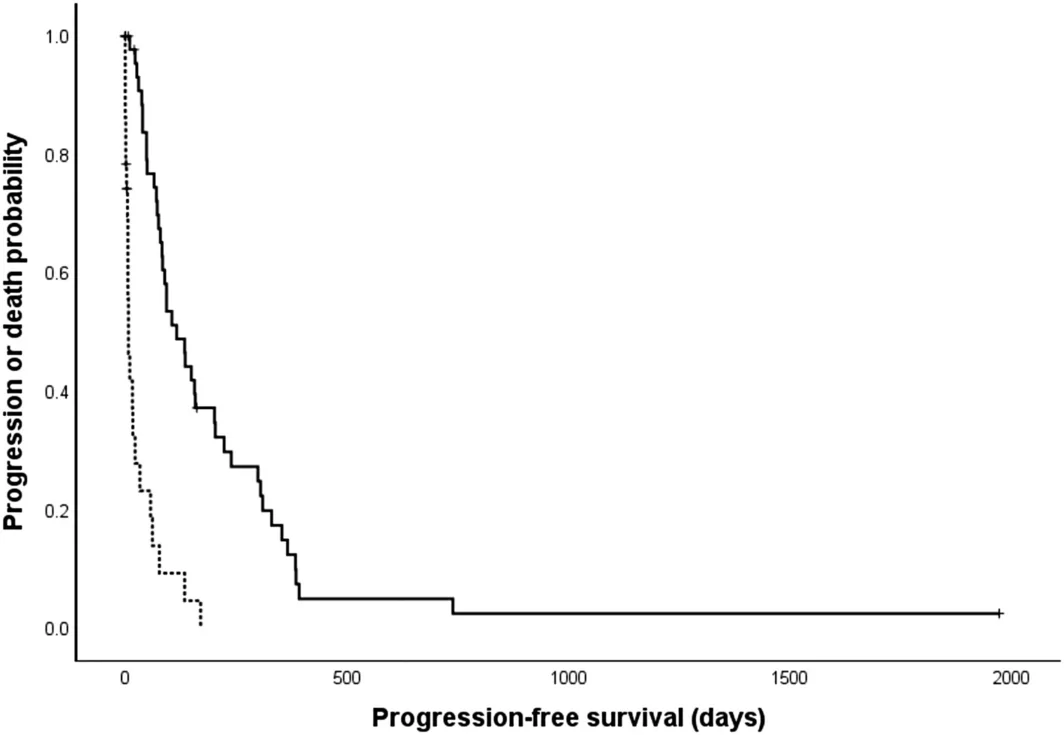
Progression‐free survival (PFS) for dogs with (solid line) and without (dotted line) treatment for pelvic osteosarcoma. Median PFS was 116 days (95% CI, 67–165) for treated dogs and 7 days (95% CI, 1–13; *p* < 0.001) for untreated dogs. Tick marks indicate censoring.

**TABLE 4:** Summary of time‐to‐event outcomes by treatment group.

Group	*n*	PFS (95% CI; range, days)	TTLP (95% CI; range, days)	TTDP (95% CI; range, days)	OS (95% CI; range, days)
All dogs	74	75 (50–100; 0–739)	93 (46–140; 3–353)	NE (−; 10–392)	79 (44–114; 0–739)
Untreated	29	7 (1–13; 0–170)			8 (0–19; 0–170)
Treated	45	116 (67–165; 10–739)	135 (79–191; 22–353)	NE (−; 30–392)	145 (104–186; 10–739)
Surgery‐based	17	72 (32–112; 10–392)	149 (10–288; 37–310)	NE (−; 384–392)	72 (48–96; 10–529)
RT‐based	29	149 (112–183; 7–739)	156 (122–190; 22–353)	NE (−; 30–366)	158 (113–203; 26–739)
Palliative RT	20	149 (92–205; 22–739)			175 (138–212; 26–739)
SBRT	8	158 (99–217; 30–385)			158 (109–208; 38–385)
Chemotherapy alone	2	105, 201^a^			114, 219^a^
All three modalities	2	149, 239^a^			150, 239^a^

*Note:* Values are medians (95% CI, range) unless indicated. The median TTDP was not estimable across groups because fewer than 50% of dogs developed distant progression before local progression or death. No statistical comparison among treatment modalities was performed due to small and overlapping subgroup sizes.

Abbreviations: CI, confidence interval; *n*, number; NE, not estimable; OS, overall survival time; PFS, progression‐free survival; RT, radiotherapy; SBRT, stereotactic body radiotherapy; TTDP, time to distant progression; TTLP, time to local progression.

^a^ Individual times shown due to minimal sample size.

Median OS for all dogs was 79 days (95% CI, 44–114), and longer survival was observed in dogs receiving any form of treatment compared with untreated dogs (Figure 2, Table 4).

**FIGURE 2:**
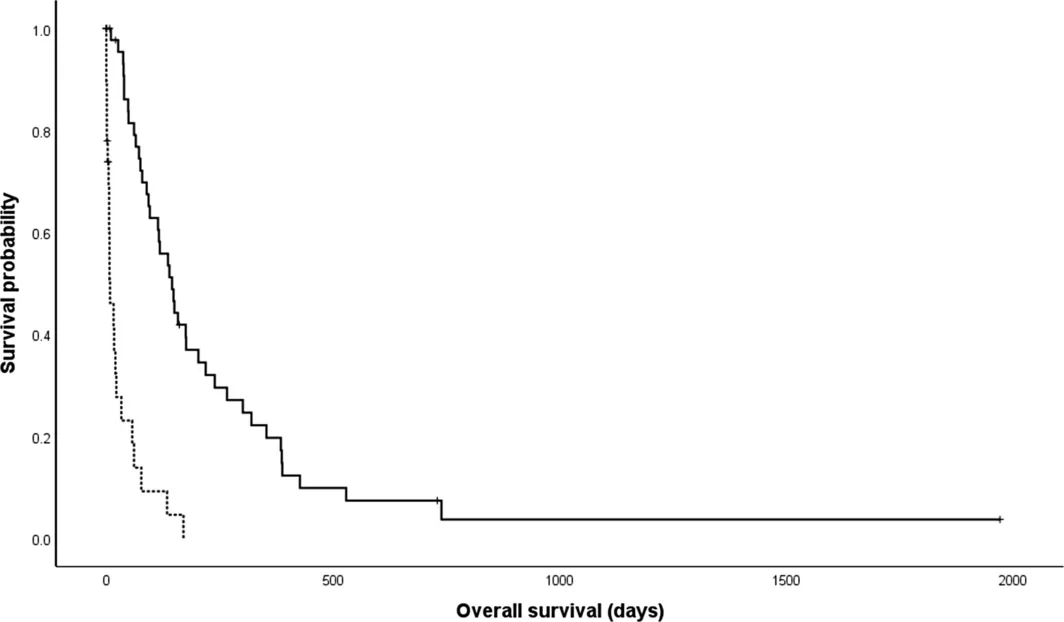
Overall survival (OS) for dogs with (solid line) and without (dotted line) treatment for pelvic osteosarcoma. Median OS was 145 days (95% CI, 104–186; *p* < 0.001) for treated and 8 days (95% CI, 0–19; *p* < 0.001) for untreated dogs. Tick marks indicate censoring.

Table 4 summarizes the time‐to‐event outcomes. No statistical comparison among treatment modalities (except treated versus untreated dogs) was performed due to small and overlapping subgroup sizes.

Twelve dogs were censored, including two dogs that were still alive at 161 and 1971 days after diagnosis, and 10 were lost to follow‐up. Most dogs (56/74, 76%) were euthanized due to tumour‐related disease, including 8/56 (14%) within 10 days of diagnosis and six (11%) at the time of diagnosis.

The reason for euthanasia was unclear in five dogs (7%), and one (1%) was euthanized due to a concurrent tumour, suspected transitional cell carcinoma of the bladder. Median follow‐up for censored dogs was 49 days (range, 0–1971).

Of the eight factors evaluated, none were significantly associated with PFS (Table 5). Cox proportional hazards regression was performed to account for potential confounding among covariates. The final model included two variables (approximately 22 events per variable), neither of which reached statistical significance.

**TABLE 5:** Factors associated with progression‐free survival.

Variable	HR	95% CI	*p*
Body weight	0.97	0.95–1.00	0.065
Time to diagnosis	1.01	1.00–1.02	0.065

Abbreviations: CI, confidence interval; HR, hazard ratio.

Using the same approach, 10 potential prognostic variables were tested for OS, and the final model retained five (8.6 events per variable; Table 6). In this model, higher body weight was associated with improved survival, whereas the use of chemotherapy was associated with an increased hazard of death. Additionally, earlier diagnosis and iliac tumour location were associated with longer survival. Dogs that had local progression had significantly shorter survival.

**TABLE 6:** Factors associated with overall survival.

Variable	HR	95% CI	*p*
Chemotherapy	2.29	1.14–4.60	0.02
Body weight	0.97	0.94–1.00	0.022
Iliac location	0.43	0.2–0.94	0.035
Time to diagnosis	1.02	1.00–1.03	0.013
Local progression	3.59	1.44–8.93	0.006

Abbreviations: CI, confidence interval; HR, hazard ratio.

## 5 Discussion

In dogs, osteosarcoma rarely affects the pelvis, and published information on this location remains limited [3, 4, 26, 27]. Our findings indicate that pelvic osteosarcoma carries a poor prognosis, with a median OS of approximately 2.5 months. While longer survival times were observed in treated dogs compared with untreated dogs, the benefit appeared modest (approximately 5 months vs. 1 week; *p* < 0.001). Unlike osteosarcoma of the long bones, where metastatic disease typically determines outcome, more than 50% of treated dogs in the present cohort succumbed to local tumour progression rather than distant metastasis [28].

Most dogs were middle‐aged to older (median, 10 years) and large‐sized (mean body weight 32 kg), consistent with previous reports [3, 17, 29]. Although higher body mass has been linked to an increased risk of developing osteosarcoma, and in several studies shorter survival in dogs > 40 kg, greater body weight in our cohort was associated with a lower hazard ratio for survival (HR = 0.97), suggesting a modest survival benefit with increasing body size [29, 30, 31, 32]. However, recently, similar survival times between small (< 15 kg) and larger dogs, and no association between body weight and the occurrence of metastases at diagnosis, have been reported [27, 33]. Given that only seven dogs (9%) weighed < 15 kg, whereas 21 dogs (28%) weighed > 40 kg, the observed association may be influenced by sampling bias. Nonetheless, the relationship is likely multifactorial and may reflect several study‐related factors rather than a true biological effect.

Lameness (84%) was the predominant clinical sign, although four dogs exhibited only signs consistent with pelvic compression (dyschezia, rectal mass effect, dysuria, or urinary obstruction). These findings emphasize the importance of including pelvic tumours among the differential diagnoses in dogs presenting with such signs. The median duration of clinical signs before diagnosis was 19 days, comparable to the 14–45 days previously described [4, 26, 27]. However, none of these previous studies were looking at pelvic osteosarcoma exclusively.

In the present cohort, longer time to diagnosis was associated with increased hazard of death, corresponding to an approximate 2% increase in hazard per day (HR = 1.02, *p* = 0.013), indicating that earlier diagnosis may improve outcomes. Complete staging was performed in approximately 70% of dogs, and suspected metastases were identified in 31% of dogs, most commonly in the lungs. This exceeded previously reported rates for appendicular (5%–15%) and axial (11%–18%) osteosarcoma in dogs, but was comparable to those for human pelvic osteosarcoma (26%–34%) [3, 4, 9, 10, 11, 12, 13, 14, 15]. These findings align with recent data showing that truncal tumours in both species have the highest odds of presenting with metastases, suggesting a shared underlying biological mechanism [27]. In humans, the higher metastatic rate has been associated with older age and delayed diagnosis, as pelvic tumours may remain clinically silent for prolonged periods [9, 10, 34].

Anatomical factors may further contribute to early metastatic spread: the axial skeleton contains red, highly vascular marrow, whereas other adult skeletal regions are composed predominantly of hypovascular yellow marrow [35, 36]. Thus, truncal osteosarcomas may lie closer to vascular tissue, facilitating early vascular invasion and hematogenous spread [27]. Post‐treatment metastasis in the current cohort was considerably lower (18%) than that reported for long bones (~90%) or mandible (~60%), but this likely reflects the substantially shorter median OS of dogs with pelvic osteosarcoma (~5 months) relative to long‐bone (~1 year) or mandibular (~1.5 years) disease [1, 17, 32, 33, 37]. Additional dogs may have developed metastases had survival not been limited predominantly by local progression. The locally aggressive nature of pelvic osteosarcoma is further supported by the significantly increased risk of death associated with local progression (HR = 3.59; *p* = 0.006), emphasizing the importance of achieving adequate tumour control.

In addition, histological data were evaluated; however, due to the limited number of dogs with available data, findings are reported descriptively. Osteoblastic (*n* = 16) and chondroblastic (*n* = 6) osteosarcomas were the most common subtypes in the present cohort, consistent with reports in people. In a recent study of human pelvic osteosarcoma, osteoblastic tumours were more frequent in older individuals (median 53.5 years), whereas chondroblastic tumours predominated in younger patients (median 22 years). Although the latter group initially appeared to have a longer survival, this difference was not maintained after age stratification [9]. The relevance of subtype and age‐related differences in dogs remains unclear [22, 38].

Long‐bone osteosarcoma is typically treated with limb amputation or limb‐sparing procedures (limb‐sparing surgery and SBRT) followed by adjuvant chemotherapy, while hemipelvectomy is generally the indicated surgical approach for the resection of pelvic tumours [14, 19, 39, 40]. Although the complex regional anatomy, the difficulty in achieving wide margins, and the associated postoperative morbidity require careful planning, a study by Bray et al. describing hemipelvectomies in a large case series reported recovery and long‐term function comparable with conventional pelvic limb amputation [19, 26]. Hemipelvectomy can be categorized as total or subtotal (removal of the entire hemipelvis or a portion thereof), and as external (removal of a pelvic segment along with the ipsilateral limb) or internal (limb‐sparing) [19, 26, 41]. External hemipelvectomy is traditionally recommended but may be less suitable in dogs with osteoarthritis, neuromuscular disease, or obesity [42, 43]. Moreover, owner perception, public attitude, and concerns regarding postoperative adaptation may also influence decision‐making [42, 44].

Limb‐sparing techniques have been described for cranial (iliac), caudal (ischial), and modified subtotal hemipelvectomies, with good functional outcomes and acceptable complication rates in appropriately selected cases [41, 42, 45, 46].

In the present cohort, surgical resection was performed in 38% of dogs, including 12 external and two internal hemipelvectomies, and three cytoreductive surgeries. In this group, the median PFS and OS remained short (72 days), likely reflecting the challenges in achieving complete surgical margins, which were obtained in only one‐third of the cases. Consistent with this, local recurrence was 47%, indicating limited durable local control. These findings differ from those of Bray et al., in which most osteosarcomas could be excised with complete margins, and recurrence occurred in 21% (6/28) of cases; that cohort, however, included a substantial number of proximal femoral osteosarcomas (19/28), in which wider margins are more readily attainable with hemipelvectomy. Similar challenges are reported in human pelvic osteosarcoma, where − although the longest survival times are achieved in surgically treated patients − the proportion of patients who undergo the procedure (~50%) is markedly lower compared to appendicular sites (~80%–90%). This has been attributed to older patient age, higher metastatic burden, extensive local tumour invasion, and greater surgical morbidity [9]. Despite these limitations, hemipelvectomy remains a viable treatment option for selected cases. In our cohort, the ilium (57%) was the most common tumour location, followed by the ischium (15%), consistent with previous veterinary and human reports [3, 4, 26]. Thus, limb‐sparing techniques may be appropriate in carefully chosen cases relative to disease location and extent, particularly in larger or older dogs, or when owners prefer limb preservation. Only three dogs in the present cohort received postoperative RT, limiting the ability to draw meaningful conclusions. However, in humans, adjuvant RT has been associated with improved survival; therefore, it may be considered when margins are incomplete or narrow to improve local control [9].

Tumour location also appeared to influence outcome: iliac tumours were modestly associated with improved survival in treated dogs (HR = 0.43, *p* = 0.035). The basis for this finding is unclear but may reflect earlier detection, as iliac tumours, being part of the weight‐bearing axis, may produce orthopaedic signs earlier than tumours in non‐weight‐bearing regions. Additionally, pubic or ischial tumours, which are positioned more ventrally and medially, may more commonly compress the rectal and urinary outflow tract. In our cohort, 11 dogs showed signs of pelvic canal obstruction; nevertheless, only four had iliac tumours, supporting that the more dorsal and lateral position of the ilium may less often compromise pelvic canal structures, potentially contributing to the better outcomes observed in this subgroup.

For dogs with inoperable pelvic osteosarcoma, palliative‐intent RT or SBRT (with definitive intent and limb‐sparing potential) may represent feasible alternatives to surgery. In the present cohort, RT represented the most common primary treatment modality, applied to 64% of the treated dogs, predominantly with palliative intent, while eight dogs received SBRT. Although the specific reasons were not investigated in the present study, this preference for RT is likely multifactorial, involving factors such as older age, pre‐existing comorbidities, feasibility of resection, presence of metastases at diagnosis, or owners' perception of postoperative recovery.

Although RT was not statistically associated with progression or survival in our study, the median OS was numerically longer with both palliative RT (175 days) and SBRT (158 days) than with surgery‐based treatment (72 days). This may represent a Type 2 error due to the small sample size. The OS observed with palliative RT was consistent with reports for long‐bone osteosarcoma (~4–10 months) [39, 47]. In contrast, survival following SBRT was shorter than expected based on recent studies (~8–12 months) [39, 48]. In our relatively small group, half (4/8) of the dogs received adjuvant chemotherapy, and only one of these dogs finished the intended protocol. In a recent study, the combination of SBRT and chemotherapy improved the outcome for dogs with appendicular osteosarcoma [48]. Bisphosphonates were administered to 22% of dogs, most often in conjunction with palliative RT, but their impact on outcome could not be determined within this study. Overall, these findings suggest that RT may represent a treatment option and should be considered, particularly when surgical resection is not feasible or declined.

Systemic therapy was administered to 40% of the treated dogs, most commonly carboplatin. Chemotherapy was associated with increased risk of death (HR = 2.29; *p* < 0.02). These results parallel the clinical course: only four dogs completed therapy, seven were euthanized for rapid progression (five after the first cycle), and two discontinued treatment because of adverse effects. This apparent negative association could reflect treatment‐selection bias rather than a true negative effect, given the small sample size and insufficient treatment duration. Conversely, two dogs that presented with metastatic disease survived 114 and 219 days, respectively, with metronomic chemotherapy. Given the aggressive biological behaviour of pelvic osteosarcoma, adjuvant chemotherapy should still be considered in dogs receiving definitive‐intent treatment, as micrometastases are likely present. Local tumour control, however, remains the more immediate challenge, emphasizing the need to optimize surgical procedure or combine it with RT when feasible. For dogs with metastatic disease, palliative RT—with or without metronomic chemotherapy and/or bisphosphonates—appears reasonable to improve quality of life.

This study has several limitations related to its retrospective design and potential inaccuracies in medical documentation. The study period of almost two decades could have influenced management and outcome, due to advances in diagnostic imaging and treatment approaches. In dogs presenting with multiple osseous lesions, definitive distinction between primary and metastatic pelvic lesions was not always possible. Dogs that were euthanised at diagnosis or shortly thereafter were also included in this study. While this reflects real‐world clinical practice, it may have introduced a negative survival bias, potentially underestimating survival times, particularly in the untreated group. Some tumours were diagnosed by cytology, raising the possibility of soft tissue sarcoma misclassification for osteosarcoma. Staging was incomplete in a subset of dogs, and metastatic disease may have been under‐detected. Most of the metastases were presumptive, as cytologic or histologic confirmation was rarely obtained. Treatment protocols were heterogeneous, and group sizes were small, limiting statistical power and increasing the potential for Type 2 errors or selection bias. Follow‐ups were inconsistent, and assessment of local progression was partly based on clinical deterioration when imaging was unavailable.

## 6 Conclusion

Pelvic osteosarcoma in dogs is an uncommon and locally aggressive tumour. In the present cohort, metastatic disease at presentation was frequently observed, and overall survival was poor. Management of this disease remains challenging, and while surgery may provide local disease control, its feasibility can be limited by tumour extent and anatomic constraints. RT, palliative or stereotactic, may represent a treatment option in cases where resection is not feasible or declined. An individualized and multimodal treatment approach may be considered in dogs with pelvic osteosarcoma.

## Funding

The authors have nothing to report.

## Ethics Statement

This retrospective multicentre study included client‐owned dogs with naturally occurring cancer and used secondary clinical data collected during routine veterinary care. Ethical review was not required by the coordinating institution or by most participating institutions under their respective institutional regulations. Where required by local regulations, ethical approval was obtained from the relevant institutional committees.

## Conflicts of Interest

The authors declare no conflicts of interest.

## Data Availability

The data that support the findings of this study are available from the corresponding author upon reasonable request.
